# Differential Autophagy Response in Men and Women After Muscle Damage

**DOI:** 10.3389/fphys.2021.752347

**Published:** 2021-11-24

**Authors:** Hui-Ying Luk, Casey Appell, Danielle E. Levitt, Nigel C. Jiwan, Jakob L. Vingren

**Affiliations:** ^1^Department of Kinesiology and Sports Management, Texas Tech University, Lubbock, TX, United States; ^2^Department of Kinesiology, Health Promotion, and Recreation, University of North Texas, Denton, TX, United States; ^3^Department of Physiology, School of Medicine, Louisiana State University Health Sciences Center, New Orleans, LA, United States

**Keywords:** cortisol, growth hormone, LC3-II/LC3-I ratio, macroautophagy, sex dimorphism

## Abstract

Following muscle damage, autophagy is crucial for muscle regeneration. Hormones (e.g., testosterone, cortisol) regulate this process and sex differences in autophagic flux exist in the basal state. However, to date, no study has examined the effect of a transient hormonal response following eccentric exercise-induced muscle damage (EE) between untrained young men and women. Untrained men (*n* = 8, 22 ± 3 years) and women (*n* = 8, 19 ± 1 year) completed two sessions of 80 unilateral maximal eccentric knee extensions followed by either upper body resistance exercise (RE; designed to induce a hormonal response; EE + RE) or a time-matched rest period (20 min; EE + REST). Vastus lateralis biopsy samples were collected before (BL), and 12 h, and 24 h after RE/REST. Gene and protein expression levels of selective markers for autophagic initiation signaling, phagophore initiation, and elongation/sequestration were determined. Basal markers of autophagy were not different between sexes. For EE + RE, although initiation signaling (*FOXO3*) and autophagy-promoting (*BECN1*) genes were greater (*p* < 0.0001; 12.4-fold, *p* = 0.0010; 10.5-fold, respectively) for women than men, autophagic flux (LC3-II/LC3-I protein ratio) did not change for women and was lower (*p* < 0.0001 3.0-fold) than men. Furthermore, regardless of hormonal changes, LC3-I and LC3-II protein content decreased (*p* = 0.0090; 0.547-fold, *p* = 0.0410; 0.307-fold, respectively) for men suggesting increased LC3-I lipidation and autophagosome degradation whereas LC3-I protein content increased (*p* = 0.0360; 1.485-fold) for women suggesting decreased LC3-I lipidation. Collectively, our findings demonstrated basal autophagy was not different between men and women, did not change after EE alone, and was promoted with the acute hormonal increase after RE only in men but not in women. Thus, the autophagy response to moderate muscle damage is promoted by RE-induced hormonal changes in men only.

## Introduction

Autophagy is a highly regulated evolutionarily conserved catabolic process responsible for sequestering and recycling dysfunctional and damaged proteins (Yang and Klionsky, 2010). Autophagosome formation is central to autophagy and requires a series of cellular processes: phagophore formation, phagophore elongation, and sequestration of the cytosolic debris through the autophagosome and delivery to the lysosome for lysosomal degradation (Mizushima, 2007). In response to stress, the activation of autophagic signaling [i.e., forkhead box O3 (FOXO3)] promotes downstream targeted autophagy-related proteins (ATG) (i.e., ATG7, ATG5), microtubule-associated protein light chain 3 LC3) (Mammucari et al., 2007), and the activation of the unc-51-like kinase (ULK1)/ATG complex followed by beclin-1 (BECN1)/vacuolar protein sorting 34 (VPS34) to regulate phagophore initiation (Russell et al., 2013). This results in the elongation of the phagophore indicated by the increase in lipidation of LC3A to LC3B controlled by ATG5 and ATG7 (Mizushima et al., 2003). Recruitment of sequestrosome 1 (p62) to LC3B in the phagophore membrane (Pankiv et al., 2007) allows for sequestration of damaged proteins in the autophagosome which then undergoes lysosomal fusion, ultimately degrading protein cargo through lysosomal proteases (He and Klionsky, 2009).

Emerging evidence shows that autophagy is increased with exercise-induced muscle damage (i.e., micro tears of the muscle fibers induced by exercise) and is critical to subsequent functional recovery and muscle regeneration (Martin-Rincon et al., 2018). In healthy young male mice, autophagosome content increased after 9 h running aiming to induce muscle damage (Salminen and Vihko, 1984). Two days after a single unaccustomed bout of RE in young men, skeletal muscle LC3-I, LC3-II, p62, and beclin1 protein content increased, but these changes were absent in aged men (Hentilä et al., 2018). Similarly, Jamart et al. (2012) reported increased *ATG4B, ATG12*, and *LC3B* gene expression after an ultra-endurance exercise in middle-aged men. Together, these results suggest that autophagy in skeletal muscle increases after damage, and this response might be delayed or impaired with age in men. Differences in autophagy may have important implications for skeletal muscle repair, regeneration, and recovery (Nichenko et al., 2016). After acute muscle damage, ATG7 knockout mice had more centralized nuclei and lower force production per unit muscle mass (Paré et al., 2017). Similarly, LC3B deficient mice had fivefold more damaged muscle fibers and smaller regenerating fibers compared to wild type 3 days after chemical-induced muscle injury (Paolini et al., 2018). Thus, autophagy signaling is critical to skeletal muscle regeneration and functional recovery after muscle damage.

Endocrine factors can increase or decrease autophagic flux. For example, testosterone and glucocorticoids appear to have opposite effects on autophagy. Castrated mice had increased FoxO3 and decreased protein kinase B (Akt) activation and increased autophagic flux (increased LC3-II/LC3-I) in skeletal muscle compared to intact mice (Serra et al., 2013; White et al., 2013). These responses were reversed by administering nandrolone decanoate (testosterone analog) to the castrated mice (Serra et al., 2013; White et al., 2013). In contrast to testosterone, the administration of dexamethasone to mice upregulated FoxO3, inhibited mammalian target of rapamycin (mTOR) activation, and increased autophagic flux in skeletal muscle (Shimizu et al., 2011). High-intensity resistance exercise (RE) induces a transient increase in circulating hormones [e.g., testosterone, growth hormone (GH), cortisol]; the role of acute RE-induced hormonal changes on skeletal muscle adaptations and satellite cell myogenic response is a topic of some controversy and have been extensively discussed (Hansen et al., 2001; Rønnestad et al., 2011; Walker et al., 2012), but these hormonal changes’ potential effect on autophagy after eccentric exercise-induced muscle damage (EE) is unknown. Importantly, men and women experience different hormonal changes in response to a bout of heavy RE, and the magnitude of hormonal increases differs between sexes (White et al., 2013). Further, the basal autophagy state in skeletal muscle differs between male and female mice (Paolini et al., 2018) and in neuronal cells in aged men and women (Congdon, 2018). However, sex differences in the autophagic response to EE have not been examined.

To date, studies have demonstrated that men and women respond differently to muscle damage, such as the degree of muscle damage and the response of cytokines (Luk et al., 2021). Autophagy and its interaction with cytokines are critical to muscle regeneration (Lira et al., 2013; Paré et al., 2017; Ge et al., 2018) and testosterone and glucocorticoids (e.g., cortisol) differentially regulate autophagic activity (Shimizu et al., 2011; Serra et al., 2013; White et al., 2013). Moreover, Rosa-Caldwell et al. suggested that men and women may preferentially favor different catabolic pathways (i.e., autophagy vs. ubiquitin proteasome pathway) (Rosa-Caldwell and Greene, 2019). Understanding sex differences in muscle damage and the subsequent intramuscular response, such as autophagy, provides foundational knowledge that can better inform advice to patients and athletes experiencing muscle damage can also be used to develop and evaluate the efficacy of interventions. Furthermore, understanding responses in young men and women could help identify aberrant responses during aging. Since the intramuscular cytokine response to muscle damage and the magnitude of the acute hormonal response to RE differ between men and women, it is possible that there is a differential autophagic response between sexes that could be altered by RE-induced hormonal changes. Thus, the purpose of this study was to determine the effects of the transient exercise-induced hormonal response on markers of autophagy in untrained young men and women after muscle damage.

## Materials and Methods

### Subjects

The study was approved by the University of North Texas Institutional Review Board (#15-351) and adhered to the Declaration of Helsinki. Written informed consent was obtained from each subject before participation in the study. The details of the study protocol and subjects demographic have been published previously (Luk et al., 2021). Briefly, due to the limited muscle samples, a subset of the sixteen healthy young untrained men (*n* = 8; 22 ± 3; 180.1 ± 5.7 cm height; 80.8 ± 15.6 kg body mass; 26.6 ± 10.3% mean body fat) and women (*n* = 8; 20 ± 1; 164.1 ± 9.0 cm height; 60.7 ± 7.8 kg body mass; 34.7 ± 9.1% mean body fat) was used for this report. To be included in the study, subjects were free from musculoskeletal injuries and self-reported no use of cigarettes, hormonal substances, or medications that could potentially confound the study variables. Further, subjects had not participated in any resistance or endurance training within the past 1 year prior to the study and engaged in less than 1 h of structured exercise per week. Women participating in the study had a normal menstrual cycle and had not used oral contraceptives for at least 3 months prior to the start of the study.

### Familiarization Visit (Anthropometric Measurements and 1-Repetition Maximum Test)

Subjects were familiarized with study procedures approximately 1 week before the first exercise session. Subjects reported to the laboratory for anthropometric measurements, familiarization with exercises, and 1-repetition maximum (1-RM) testing (Men: bench press: 64.99 ± 18.27 kg, bench row: 56.83 ± 10.93 kg, seated overhead press: 39.96 ± 10.61 kg. Women: bench press 24.67 ± 7.93 kg, bench row: 27.71 ± 7.62 kg, seated overhead press: 16.64 ± 6.71 kg). After height and weight measurement, body composition was measured using Dual-energy X-ray absorptiometry (DXA; GE Lunar Prodigy, Madison, WI, United States). Then, subjects completed a standardized dynamic warm-up, were familiarized with the unilateral eccentric knee extension protocol for each leg using only light effort to avoid muscle damage, and were instructed in proper technique for performing the free weight upper body exercises (bench press, bench row, and seated overhead press). Once subjects demonstrated proper technique, their 1-RM for the bench press, bench row, and overhead press exercises were measured.

### Exercise Sessions

**FIGURE 1:**
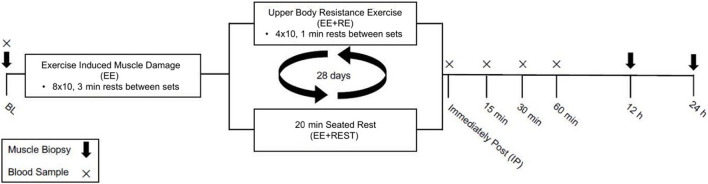
Schematic diagram of the study design.

The details of the exercise protocol have been described previously (Luk et al., 2019). The study employed a within-subjects design in which each subject completed both experimental conditions and thus served as their own control. Briefly, subjects completed two exercise visits with an identical unilateral maximal effort eccentric knee extension (i.e., force was only applied during the muscle lengthening phase of the action) aimed to induced muscle damage without drastically increasing circulating RE-induced hormones. Immediately after the maximal effort unilateral eccentric knee extension exercise, subjects either completed 20 min heavy upper body RE consisting of four sets of 10 repetitions of bench press, bench row, and seated overhead press exercises with 1 min rest between sets and exercises, performed at 80% of 1-RM, aimed to induce hormonal changes (EE + RE) or seated time-matched rest for 20 min (EE + REST). Further, the heavy upper body RE increased circulating testosterone, GH, cortisol. These increases were absent in EE + REST despite having performed unilateral eccentric knee extensions (Spiering et al., 2009). Muscle biopsies were obtained from the vastus lateralis of the exercising leg before (BL) and at 12 (12 h) and 24 (24 h) hours after the exercise session. For women, the exercise sessions were conducted during the early follicular phase of two consecutive menstrual cycles. To determine the menstrual cycle phase, women participants were instructed to self-report the onset of menses; then exercise sessions were scheduled 2–7 days after the onset of menses to control for hormonal variation throughout the cycle. To maintain consistency of procedures between sexes, the two exercise sessions were separated by approximately 28 days for all participants (Figure 1).

To confirm muscle damage, serum creatine kinase (CK) activity was measured immediately after the maximal effort unilateral eccentric knee extension exercise (IMD; CK is not expected to differ from baseline at this time point) and at 12 h and 24 h after completion of the upper body RE or 20 min rest. The CK results have been reported previously (Luk et al., 2021). Briefly, CK was significantly greater (1.6-fold) at 12 h in men than women. For men, CK was significantly increased at 12 h by 2.58-fold and 24 h by 4.45-fold from IMD. For women, CK was significantly increased at 12 h by 1.184-fold and 24 h by 2.101-fold from IMD. Meanwhile, there was no difference in CK between conditions. The difference between sexes was expected as previous studies demonstrated that men exhibited a greater CK response than women at 24–72 h after EE (Oosthuyse and Bosch, 2017).

Furthermore, our previously published results had shown that the upper body RE protocol was able to induce a greater magnitude of increase in testosterone, GH, and cortisol concentration for EE + RE (Luk et al., 2019). Briefly, the area under curve (AUC) for testosterone was significantly 1.3-fold greater for EE + RE than EE + REST in men, whereas there was no difference for women between conditions. Furthermore, AUC of testosterone was significantly greater in men than in women for EE + RE (10.3-fold) and EE + REST (9.8-fold). The AUC of GH was significantly 4.3-fold greater for EE + RE than EE + REST for men, whereas there was no difference for women between conditions. Furthermore, AUC of GH was significantly 2.8-fold greater in men than in women for EE + RE with no sex difference for EE + REST. The AUC of cortisol was significantly 1.8-fold greater for EE + RE than EE + REST with no differences between sexes.

For each exercise visit, subjects reported to the laboratory in the morning after a 10–12 h overnight fast (except for water). Hydration status was assessed using a refractometer at the beginning of all visits. After 10 min of seated rest, a cannula was inserted in a superficial vein of the forearm and pre-exercise blood samples were collected. The cannula was kept patent with sterile saline. After blood collection, muscle samples were obtained from the vastus lateralis of the leg to be exercised under local anesthesia (1% lidocaine without epinephrine) using a single-use Pro-Mag 14-gauge muscle biopsy needle (Angiotech, Gainesville, FL, United States). Participants completed the standardized dynamic warm-up followed by unilateral maximal effort eccentric knee extensions (eight sets of 10 repetitions at maximal effort with 3 min of passive rest between sets). Eccentric leg extensions were performed before upper body RE or seated rest to avoid any adverse effects of fatigue on eccentric knee extension effort. The order of exercising leg (left or right) for the knee extension protocol and order of condition (EE + RE or EE + REST) were counterbalanced and assigned using randomization.

Subjects then either rested in a seated position for 20 min (EE + REST) or performed a 20 min upper body RE protocol (EE + RE). The upper body RE protocol consisted of four sets of 10 repetitions of bench press, bench row, and seated overhead press exercises with 1 min rest between sets and exercises. The initial load for each exercise was set at 80% of 1-RM. When a subject could not finish a repetition, assistants provided minimal assistance to help complete the concentric portion of the remaining repetitions for that set and the load was then reduced accordingly to allow participants to complete the remaining sets for that exercise. Blood samples were collected immediately after EE (IMD, immediately (IP), and 15 (15 min), 30 (30 min), and 60 (60 min), 12 h, and 24 h after the 20 min upper body exercise or rest period. Subjects returned to the laboratory for muscle sample collection (from the same leg as sampled for BL) at 12 h, and 24 h after their exercise session reporting time (30 min). Three separate sites for muscle sampling were 3 cm apart on the vastus lateralis to avoid confounding influences of local immune/inflammatory responses. Once each sample was obtained, it was immediately flash frozen in liquid nitrogen.

To minimize potential confounding variables from meal intake, subjects were instructed to record the time of the meal, type, and amount of food and drink consumed following their first exercise session, which included lunch, snack, and dinner for that day. Subjects had finished dinner 3 h prior to the 12 h visit and were not allowed to consume any food or drink (except water) until the morning after the 24 h visit. The exact diet (mealtime, type, and amount of food and drink) was followed for the second exercise session. Further, subjects were not allowed to consume non-steroidal anti-inflammatory drugs and/or alcohol 48 h prior to and 24 h after each exercise session.

### RT-qPCR Analysis

Details of muscle tissue homogenization and RNA isolation have been published previously (Luk et al., 2021). After removing visually apparent fascia and adipose, RNA was isolated from flash frozen muscle samples (∼30 mg) using the RNeasy Fibrous Tissue Mini Kit (Cat. # 74704, Qiagen, Germantown, MD, United States) with the recommended standardized protocol. cDNA was synthesized from 1 μg of total RNA using Iscript reverse transcription kit (Bio-Rad MP3, Bio-Rad Laboratories, Hercules, CA, United States). Real-time PCR amplification experiments and calculations of relative expression levels were performed following the user manual # 2 ABI PRISM70500 Sequence Detection System (Applied Biosystems, Waltham, MA, United States) with iTag SYBR Green Supermix (Bio-Rad MP3, Bio-Rad Laboratories, Hercules, CA, United States). Pre-designed assays were obtained from Integrated DNA Technologies with beta 2-microglobulin (*B2M*) as the internal control (Table 1). Duplicate biological replications and triplicate technical replications were performed for each assay. Data were then analyzed using the relative mRNA method and expressed as fold change vs. the average expression in men in the EE + REST condition at BL.

**TABLE 1:** List of RT-qPCR assays and their corresponding genes.

**No**	**IDT assay ID**	***Gene***	**Protein ID**
1	Hs.PT.58.26215470	*AKT*	NM_001014431
2	Hs.PT.58.2898629	*ATG5*	NM_004849
3	Hs.PT.58.1332116	*ATG7*	NM_001136031
4	Hs.PT.58.504143	*BECN1*	NM_003766
5	Hs.PT.58v.18759587	*B2M*	NM_004048
6	Hs.PT.58.25522530.g	*FOXO3*	NM_001455
7	Hs.PT.58.27450194	*LC3A*	NM_181509
8	Hs.PT.58.27295455.g	*LC3B*	NM_022818
9	Hs.PT.58.24456023	*MTOR*	NM_004958
10	Hs.PT.58.39829257	*p62/SQSTM1*	NM_003900
11	Hs.PT.58.27473616	*ULK1*	NM_003565

### Western Blot Analyses

A detailed description of muscle homogenization and western blot analyses has been published previously (Luk et al., 2019). Briefly, flash frozen muscle samples were homogenized in 10 μL/mg muscle of ice-cold RIPA buffer (Sigma Aldrich, St. Louis, MO, United States) containing 1X protease inhibitor (1:50; complete, Mini, EDTA-free Protease Inhibitor Cocktail, Sigma Aldrich, St. Louis, MO, United States) on ice using a handheld homogenizer (Argos Technologies, Vernon Hills, IL, United States). Homogenized muscle samples were agitated at 4°C, then centrifuged at 15,000 × *g* at 4°C for 20 min. The supernatant was collected, analyzed for total protein concentration using microplate spectrophotometry (Epoch, Bio-Tek, Winooski, VT, United States), and stored at −80°C until protein analysis.

Total protein was diluted in 1X Tris Buffered Saline (TBS), 2X Laemmli buffer, and 1 M dithiothreitol (Bio-Rad MP3, Bio-Rad Laboratories, Hercules, CA, United States) and 40 μg of protein was separated in precast 4–20% Mini-Protean TGX gels (Bio-Rad MP3, Bio-Rad Laboratories, Hercules, CA, United States) using SDS-PAGE at 120 V. Separated protein was then transferred (70 V, 4°C) to PVDF membranes for immunoblotting. All samples from one subject (three timepoints and two conditions) were loaded on the same gel and duplicate gels were used. Further, a set of duplicate gels were cropped according to the molecular weight of a man and a woman were transferred to the same PVDF membrane to account for sex comparison.

Immunoblotting was carried out using rabbit monoclonal antibodies against LC3A/B (1:5,000; Cell Signaling Technology Cat# 12741, Lot 4 RRID: AB_2617131, Danvers, MA, United States), p62 (1:2,000; Novus Cat# NBP1-48320, Lot F RRID: AB_10011069, Englewood, CO, United States), and normalized to GAPDH (1:5,000; Santa Cruz Biotechnology Cat# sc-365062, Lot# C2816 RRID: AB_10847862, Dallas, TX, United States). Membranes were blocked in 5% w/v non-fat dry milk in TBS containing 0.1% Tween-20 (TBST) and room temperature for 1 h, incubated overnight at 4°C with primary antibodies diluted in TBST with 3% non-fat dry milk, and subsequently incubated with secondary antibody conjugated to horseradish peroxidase diluted 1:10,000 in TBST with 5% non-fat dry milk at room temperature for 1 h. Stained protein bands were visualized using a chemiluminescent substrate (WesternBright Sirius HRP substrate, Advanta, Menlo Park, CA, United States) and the C-Digit imaging system (Li-Cor, Lincoln, NE, United States). Band densitometry was performed using Image Studio Digits Ver 5.0 (Li-Cor, Lincoln, NE, United States).

### Statistical Analysis

SPSS (IBM version 26; Armonk, NY, United States: IBM Corp) and G^∗^Power (version 3.1; Heinrich Heine University Düsseldorf, Düsseldorf, Germany) was used for all statistical analyses. Data for each variable were evaluated to determine if the assumptions (e.g., normality, sphericity) for parametric statistics were met. Log10 transformation was used where the assumption of normality was violated. All data were log transformed for analyses and presented as raw data. Gene and protein expression levels were analyzed using a three-way ANOVA (time × sex × condition) with repeated measures on condition and time. For each significant main or interaction effect, Bonferroni *post hoc* test was used for pairwise comparisons.

Pearson correlation coefficients were generated to determine associative relationships between the mean changes in muscle LC3-I, LC3-II, p62 protein expression, and LC3-II/LC3-I from BL to 12 h and BL to 24 h and previously published hormonal data (testosterone, GH, and cortisol area under curve: AUC). The mean changes for the expression of these proteins were used for analyses because this would provide information on the overall autophagy protein response within 24 h after muscle damage. The significance level for this study was set at *p* < 0.05. Data are reported as mean ± SD.

## Results

To evaluate autophagy, gene expression for selected markers of autophagy initiation signaling (*FOXO3, AKT, mTOR*), phagophore initiation (*ULK1, ATG5, BECN1*), and elongation and sequestration (*ATG7, LC3A, LC3B, p62*), as well as protein expression for LC3-I, LC3-II, and p62 were analyzed in skeletal muscle samples collected at BL, and 12 h and 24 h after the upper body RE or time-matched rest. There was no significant (*p* > 0.05) difference observed in the expression of these selective autophagy markers between young men and women.

A bout of maximal effort unilateral eccentric knee extension exercise followed by 20 min seated rest (EE + REST) did not significantly affect the gene expression at 12 h and 24 h for selective markers of autophagy initiation signaling, phagophore initiation, elongation, and sequestration in young men and women. With the lack of change in the transcript abundance on these selective molecular markers, our findings of no changes in LC3-I, LC3-II, and p62 protein expression provide further evidence for a lack of an autophagy response in both men and women after EE + REST. The role of autophagy in muscle regeneration has been shown, but our findings suggest that moderate EE alone does not change muscle autophagy in the first 24 h after damage.

**FIGURE 2:**
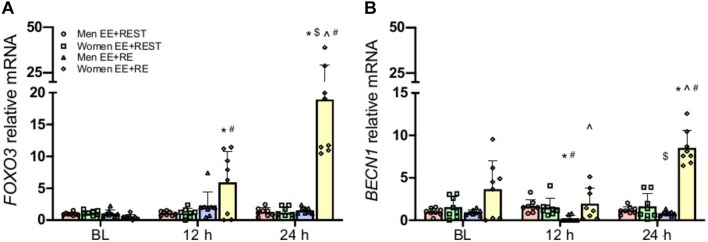
Gene expression analysis for *FOXO3* **(A)**, *BECN1* **(B)**. Data was normalized to Men EE + REST at BL. A sex × condition × time interaction was observed for *FOXO3* and *BECN1.* Values are mean ± SD (men: *n* = 8; women: *n* = 8). *p < 0.05 vs. BL. $*p* < 0.05 vs. 12 h. ∧*p* < 0.05 vs. men. #*p* < 0.05 vs. EE + REST.

With mounting evidence on the regulating role of hormones (e.g., testosterone, cortisol, etc.) on autophagy and the well-established endogenously increases in these hormones from a bout of heavy RE, it is important to evaluate if the sex-specific hormonal acute changes from RE can alter the autophagy response to EE. To accomplish this, a heavy upper body RE was implemented immediately after the EE to induce an acute transient hormonal change. For autophagy signaling initiation, our findings suggested that the addition of an upper body RE after the maximal effort eccentric knee extension exercise (EE + RE) did not significantly change *AKT* and *mTOR* gene expression. Meanwhile, *FOXO3* gene expression was significantly increased at 12 h (*p* = 0.0410; 10.79-fold) and 24 h (*p* < 0.0001; 36.68-fold) from BL and at 24 h (*p* = 0.0160; 2.19-fold) from 12 h in women for EE + RE. These increases resulted in a significantly greater *FOXO3* gene expression in women compared to men (*p* < 0.0001; 12.4-fold) for EE + RE at 24 h, and for EE + RE compared to EE + REST at 12 h (*p* = 0.0410; 5.4-fold) and 24 h (*p* < 0.0001; 15.7-fold) in women (Figure 2A).

With the increase in the *FOXO3* in women, its downstream phagophore initiation marker *BECN1*, the autophagy promoting gene, also had a significant increase at 24 h (*p* = 0.0380; 1.33-fold) from BL for EE + RE which led to a significantly (5.2-fold) greater *BECN1* for EE + RE than EE + REST at 24 h in women. On the contrary, *BECN1* was significantly decreased at 12 h (*p* = 0.0020; 0.8-fold) from BL and increased at 24 h (*p* < 0.0001; 3.36-fold) from 12 h for EE + RE in men, which led to a significantly (*p* < 0.0001; 9.0-fold) lesser *BECN1* for EE + RE than EE + REST at 12 h. These changes resulted in a significantly greater *BECN1* gene expression for EE + RE in women than men at 12 h (*p* = 0.0010; 10.5-fold) and 24 h (*p* < 0.0001; 10.6-fold) (Figure 2B). Meanwhile, there was no significant difference for *ULK1* and *ATG5* gene expression.

**FIGURE 3:**
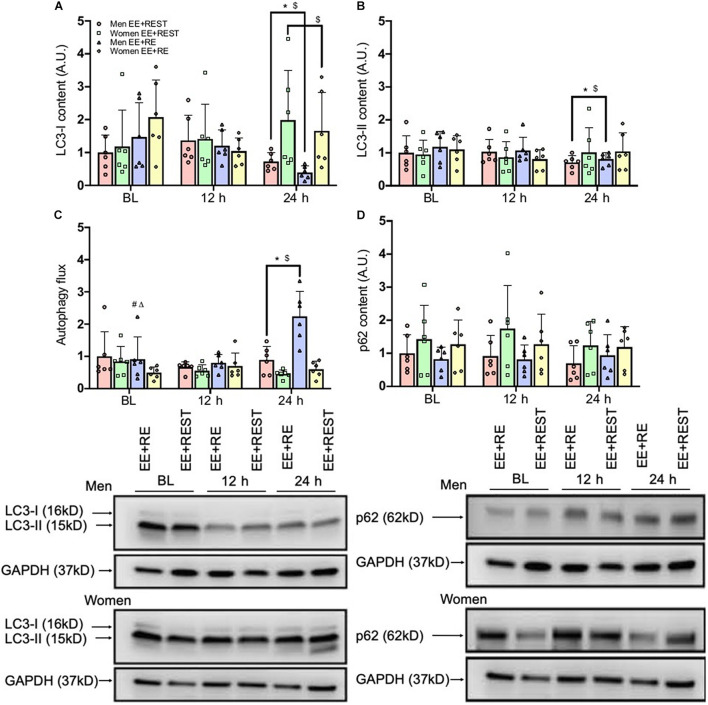
Protein content results for LC3-I **(A)**, LC3-II **(B)**, Autophagic flux (LC3-II/LC3-I) **(C)**, and p62 **(D)**. Data was normalized to the Men EE + REST at BL. Representative western blots (below figures) display BL, 12 h, and 24 h loading protein contents for LC3-I and LC3-II and p62 and its corresponding GAPDH (loading control) for an individual subject between conditions (EE + RE and EE + REST) run on the same gel for men and women. Values are mean ± SD (men: *n* = 6; women: *n* = 6). MW, molecular weight (kD). *p < 0.05 vs. BL (collapsing for condition). $*p* < 0.05 vs. 12 h (collapsing for condition). #*p* < 0.05 vs. EE + REST (collapsing for time). Δ*p* < 0.05 vs. women (collapsing for time).

Despite the increased transcript abundance on *FOXO3* and *BECN1* in women for EE + RE, this transcriptional signal did not translate into an increase in phagophore elongation and sequestration. Our finding on autophagy flux (LC3-II/LC3-I protein ratio) demonstrated a significantly (*p* = 0.0010; 2.2-fold) greater flux for men than women in EE + RE with no sex difference in EE + REST. Furthermore, the autophagy flux was significantly (*p* = 0.0020; 1.5-fold) greater for EE + RE than EE + REST in men, whereas no significant difference between conditions was observed for women (Figure 3C).

Regardless of the conditions, results from the protein analysis suggested that in men both LC3-I and LC3-II were significantly decreased at 24 h from BL (LC3-I: *p* = 0.0090; 0.547-fold; LC3-II: *p* = 0.0410; 0.306-fold) and 12 h (LC3-I: *p* = 0.0140; 0.565-fold; LC3-II: *p* = 0.0430; 0.279-fold). In contrast, in women LC3-I was significantly increased at 24 h (*p* = 0.0360; 0.485-fold) from 12 h with no changed in LC3-II from BL (Figures 3A,B). Further, for men, autophagy flux was significantly increased at 24 h from BL (*p* = 0.0010; 0.637-fold) and 12 h (*p* < 0.0001; 0.11-fold), which resulted in a significantly greater autophagy flux at 24 h for men than women (*p* < 0.0001 3.0-fold) (Figure 3C). Lastly, there was no significant difference in p62 protein expression (Figure 3D).

**FIGURE 4:**
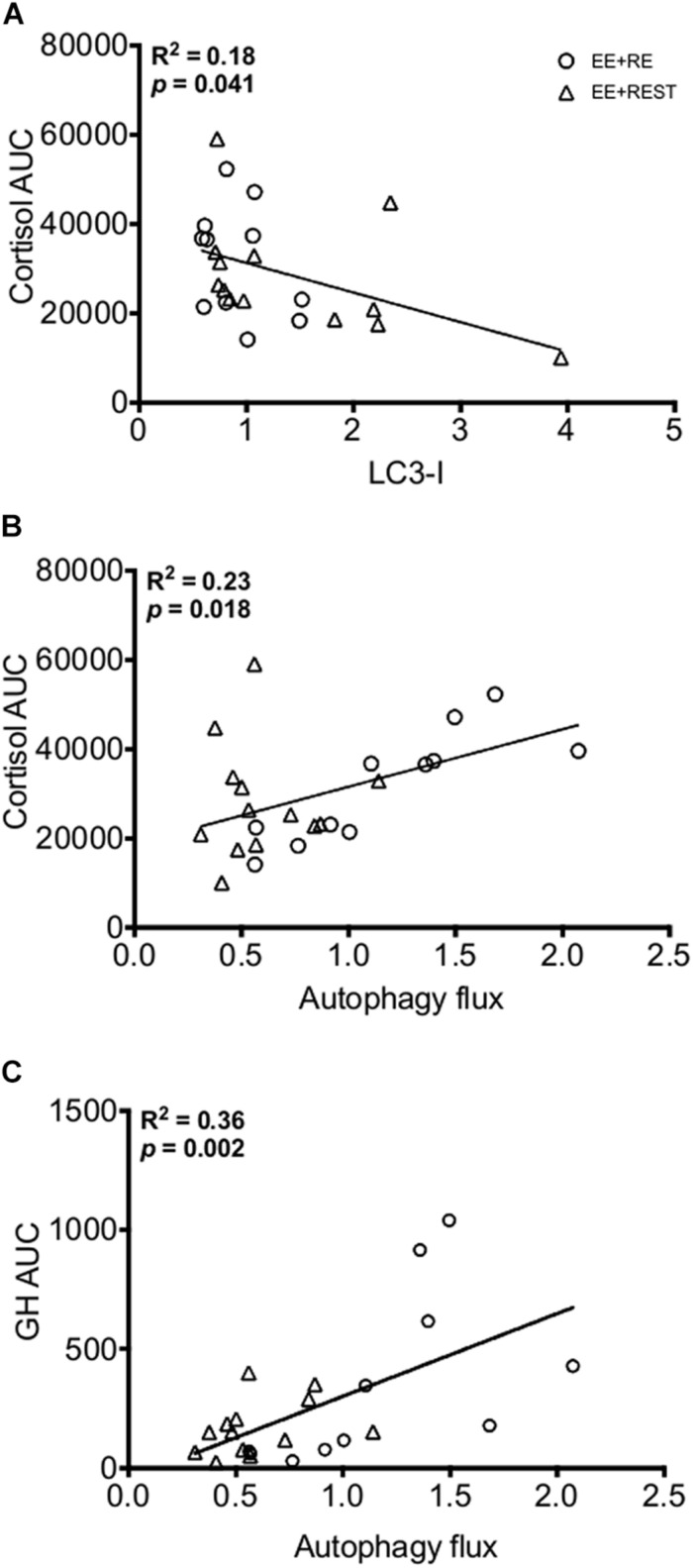
Bivariate correlation analysis (EE + RE and EE + REST) for LC3-I content to Cortisol area under curve (AUC) **(A)**, Autophagy flux to Cortisol AUC **(B)**, and Autophagy flux to Growth Hormone (GH) AUC **(C)**.

Independent of sex, our findings indicated that LC3-I protein content was significantly decreased at 24 h (*p* = 0.0090; 0.421-fold) from BL with an increase in autophagy flux at 24 h from BL (*p* = 0.0090; 1.02.1-fold) and 12 h (*p* = 0.0090; 0.897-fold) for EE + RE. To further evaluate the potential associative relationship between the RE-induced hormone changes and the autophagy markers. Bivariate correlational analysis was performed. Our correlative results showed that the average LC3-I protein content at 12 h and 24 h was significantly negatively associated with area under curve (AUC) of cortisol (*p* = 0.0410; *R*^2^ = 0.18) (Figure 4A) and the average autophagy flux at 12 h and 24 h was significantly positively associated with AUC of cortisol (*p* = 0.0180; *R*^2^ = 0.23) (Figure 4B) and AUC of GH (*p* = 0.0020; *R*^2^ = 0.36) (Figure 4C).

## Discussion

The major findings of this study are that the addition of a resistance exercise (RE)-induced a transient increase in hormones immediately after eccentric exercise-induced muscle damage (EE) increased autophagy initiation signaling genes, LC3-I lipidation (LC3-I protein decreased), and autophagic flux, whereas, in response to EE alone (no transient increase in hormones), autophagy did not change over the initial 24 h recovery period. However, these differences between conditions were mainly driven by the effect of sex. When an upper body RE was implemented following EE on the lower extremity, despite an increase in initiation signaling (*FOXO3*) and key autophagy-promoting (*BECN1*) genes observed for women only, the autophagic flux did not change for women and was lower than for men. Furthermore, when disregarding the effect of hormonal changes, results indicated an increase in LC3-I lipidation and autophagosome degradation (LC3-II protein decreased) for men and an LC3-I protein accumulation for women. Notably, in the absence of muscle damage (baseline), there was no difference in autophagy genes, proteins, and autophagy flux in skeletal muscle between sexes. Combined, our results illustrated that upper body RE-induced transient increases in hormones immediately after lower extremity EE, autophagy was promoted for men but not for women. Given the importance of autophagy in muscle repair, sex differences including those for hormonal responses could potentially influence muscle regeneration and restoration of function after damage.

Although autophagy is required by skeletal muscle for homeostatic functions, including regeneration after damage (Lee et al., 2019), it is not known if muscle damage alone can induce autophagy, or whether another signal is required to alter autophagy response for muscle recovery. In this study, eccentric exercise-induced muscle damage (eight sets of 10 reps maximal knee extension exercise) was used to induce moderate muscle damage (Luk et al., 2019). To date, studies in exercise-induced autophagy used either traditional RE (i.e., induce hormonal response) or prolonged endurance running (i.e., induce energy deficit), and hormones and energy state can each induce autophagy. Since direct measurement of autophagy activity is not feasible in humans, LC3-I, LC3-II, and p62 protein have been used as indirect markers. Engulfing materials by forming autophagosomes requires the lipidation of LC3-I to LC3-II (an anchoring protein residing in the autophagosome) and therefore, the degradation of autophagosome will also degrade LC3-II and p62 (a surrogate marker of lysosomal degradation) (Fritzen et al., 2016; Klionsky et al., 2016). For example, a decrease in LC3-II protein is considered as the decrease in autophagosome content; however, without a decrease in p62 protein, we would not be able to determine if this decrease is the result of a decrease in autophagosome formation (decrease autophagy flux) or an increase in autophagosome degradation (increase autophagy flux) (Rubinsztein et al., 2009).

To date, this is the first study to examine if the autophagy response to muscle damage differs between sexes and whether that response is altered by the transient exercise-induced increase in circulating hormones. Our results demonstrated that when collapsing for condition, the increased in LC3-I content at 24 h from 12 h with no difference from baseline for women suggested that autophagy capacity was the same as baseline. In addition, with no change in the LC3-II, p62, and LC3-II/LC3-I indicated that the balance between the rate of engulfing damaged protein by autophagosomes and the rate of degrading autophagosomes was no difference (Klionsky et al., 2016) at the first 24 h after muscle damage. On the contrary, for men, there was decreased content for LC3-I and LC3-II regardless of the condition in which suggested that the rate of autophagosome degradation exceed the rate of autophagosome formation (Tanida et al., 2005; He and Klionsky, 2009). Since autophagy is critical to tag and to engulf damaged protein for degradation, the different autophagy responses between sexes could be due to a greater demand for degrading damaged protein in men. In accordance, we had previously reported that with the same relative total work performed, CK was greater for men than women at 12 h and with a similar trend at 24 h (Luk et al., 2021). The degree of muscle damage might, at least partly, explain the increase in autophagosome degradation observed in men but not in women at 24 h. With the importance of autophagy in muscle regeneration, the lack of change in autophagy for women could be interpreted to negatively impact muscle recovery. However, unaltered autophagy markers could suggest that basal autophagy activity was sufficient to address the muscle damage in women, or as has been previously reported, that women could have preferentially relied upon the UPS over the autophagy pathway for protein degradation (Rosa-Caldwell and Greene, 2019). It is noteworthy to mention that CK was not different between conditions for men or women (Luk et al., 2021), yet a greater overall autophagy flux in men for EE + RE than in men for EE + REST and greater than in women overall suggested that other factors, besides the degree of muscle damage, can affect the autophagy response.

In response to muscle damage, both autophagy and cytokines are critical for muscle regeneration (Philippou et al., 2012; Call and Nichenko, 2020) and their interactions have been previously demonstrated in cell lines (Keller et al., 2011; Lee et al., 2011; Iovino et al., 2012). Evidence demonstrates that TGF-β and TNF-α promoted autophagy in L6 (rat), C2C12 (mouse), and primary human myoblasts (Keller et al., 2011; Lee et al., 2011; Iovino et al., 2012), whereas IL-10 inhibited autophagy in lymphocytes and macrophages (Park et al., 2011; Buchser et al., 2012). These findings might provide insights toward our differential autophagic flux between sexes. In a previous report, we have shown that, for men, TGF-β increased at 12 h for EE + RE but did not change for EE + REST. In addition, TNF-α increased at 24 h for both conditions; however, the increase was primarily driven by EE + REST, whereas IL-10 increased at 12 h and 24 h for EE + REST only (Luk et al., 2021). On the contrary, there was no change in these cytokines for women (Luk et al., 2021). We speculated that the differential autophagy response in men between conditions could be, at least partly, explained by the cytokines’ response to muscle damage (Levitt et al., 2017; Suzuki, 2019).

The main difference between the two conditions in the present study was the transient increase in circulating factors (e.g., testosterone, GH, and cortisol) and these results have been published previously (Luk et al., 2019). Briefly, for women, testosterone and GH area under the curve (AUC) from immediately post the exercise protocol to 60 min post-RE or REST was no different between conditions. For men, testosterone and GH AUC were 31 and 324% greater in EE + RE than EE + REST. For men, GH AUC was 184% greater than women. Lastly, for both men and women, cortisol AUC was 79% greater in EE + REST than EE + RE (Luk et al., 2021). Our GH results are consistent with Kraemer et al. (1991) who, despite, demonstrated no difference between men and women after a full body RE at a given time point; the magnitude of increase was in fact greater in men than in women. The relationship between cortisol and autophagy markers observed in this study were consistent with previous findings on the stimulatory role of cortisol, induced by caloric restriction or exposed to cortisol analog, on autophagy markers in human and cell culture models (Troncoso et al., 2014; Yang et al., 2016). This study is the first to demonstrate that elevated cortisol induced by RE was positively associated with autophagic flux and negatively associated with LC3-I protein content. Similar to cortisol, RE-induced GH demonstrated a positive association with autophagic flux. Although previous study in cancer cells had demonstrated that GH receptors signaling activated non-canonical autophagy in cancer cells (Velázquez et al., 2016; Zhang et al., 2018), evidence of the role of GH signaling on skeletal muscle autophagy is lacking. Despite the muscle sampling time points did not coincide with the change in hormones, it has been demonstrated the downstream effect of hormone does not occur until later and after the peak in concentration has subsided (Nielsen et al., 2008). Thus, with a greater GH AUC for men than women in EE + RE and with no difference in cortisol AUC, and the finding that GH was positively correlated with autophagic flux, that the differential autophagic flux between sexes for EE + RE could be, at least partly, driven by GH.

In summary, previous studies have demonstrated the crucial role of autophagy in muscle regeneration and the role of sex and stress hormones in regulating autophagy. To our knowledge, this is the first study to investigate select molecular markers of autophagy and their upstream regulators in untrained young men and women after EE and RE-induced hormonal changes. Our results illustrate an overall sex difference in autophagy after exercise where men had a higher autophagy flux than women. Based on these findings, we suggest that the addition of a bout of RE immediately after muscle damage could alter the autophagy response in men. Factors such as GH and cortisol could likely play a role. However, in the complex physiological environment, it is difficult to isolate a specific factor affecting the expression of these markers of autophagy and their upstream regulators.

## Data Availability Statement

The original contributions presented in the study are included in the article/Supplementary Material, further inquiries can be directed to the corresponding author/s.

## Ethics Statement

The studies involving human participants were reviewed and approved by University of North Texas Institutional Review Board (#15-351). The patients/participants provided their written informed consent to participate in this study.

## Author Contributions

H-YL and JV conceived and designed the research. H-YL, DL, and JV conducted the data collection. H-YL, CA, and NJ contributed in the sample and data analyses. H-YL and CA wrote the manuscript. All authors read and approved the manuscript.

## Conflict of Interest

The authors declare that the research was conducted in the absence of any commercial or financial relationships that could be construed as a potential conflict of interest.

## Publisher’s Note

All claims expressed in this article are solely those of the authors and do not necessarily represent those of their affiliated organizations, or those of the publisher, the editors and the reviewers. Any product that may be evaluated in this article, or claim that may be made by its manufacturer, is not guaranteed or endorsed by the publisher.

## Abbreviations

AKT: protein kinase B
ATG: autophagy-related protein
AUC: area under curve
BECN1: beclin-1
BL: baseline
CK: creatine kinase
EE: eccentric exercise-induced muscle damage
ERK: extracellular signal-regulated kinase
FOXO3: forkhead box (Fox) O3
GH: growth hormone
LC3: microtubule-associated protein 1A/1B-light chain 3
LPS: lipopolysaccharide
MAPK: mitogen-activated protein kinase
mTOR: mammalian target of rapamycin
RE: resistance exercise
p62/SQSTM1: sequestosome 1
ULK-1: unc-51-like kinase
VPS34: vacuolar protein sorting 34.
