# Supplementary material for: Differential Autophagy Response in Men and Women After Muscle Damage
Source: Front Physiol. 2021 Nov 24;12:752347. doi: 10.3389/fphys.2021.752347 (PMC8652069; doi:10.3389/fphys.2021.752347)
Supplement: Supplementary file 1 [file Data_Sheet_1.docx]

Supplementary Material

## Supplementary Figure

## Supplementary Figure

##

**Supplementary Figure 1. Full Western Blot Image**. The western blots display an example of LC3-I & LC3-II (a) and p62 (b). In a 4-20% SDS-PAGE gel, from left to right, wells represent EE+RE at BL, EE+REST at BL, EE+RE at 12-h, EE+REST at 12-h, EE+RE at 24-h, EE+REST at 24-h. Men and women duplicated (A and B) gels were place on the same membrane PVDF membrane for immunoblotting.
